# Comparison of Essential Oil Components and *In Vitro* Antioxidant Activity of *Zanthoxylum nitidum* from Different Parts

**DOI:** 10.3390/plants14081194

**Published:** 2025-04-11

**Authors:** Yang Yang, Yanqun Li, Hanjun He, Leilei Yang, Jiaxin Zeng, Mei Bai, Hong Wu

**Affiliations:** 1 School of Pharmaceutical Sciences, Hunan University of Medicine, Huaihua 418000, China; hnum_yy@163.com; 2 Guangdong Laboratory for Lingnan Modern Agriculture, College of Life Sciences, South China Agricultural University, Guangzhou 510642, China; 3 Guangdong Key Laboratory for Innovative Development and Utilization of Forest Plant Germplasm, College of Forestry and Landscape Architecture, South China Agricultural University, Guangzhou 510642, China; 4 Laboratory of Southern Subtropical Plant Diversity, Fairylake Botanical Garden, Shenzhen & Chinese Academy of Sciences, Shenzhen 518004, China

**Keywords:** *Zanthoxylum nitidum*, essential oils, different parts, *in vitro* antioxidant activities, hierarchical cluster analysis (HCA), principal component analysis (PCA)

## Abstract

*Zanthoxylum nitidum* is a traditional Chinese herb, but limited information is available concerning its composition and pharmacological effects of essential oils from different parts of *Z. nitidum*. This study examined the composition and *in vitro* antioxidant activity of essential oils from different parts of *Z. nitidum* in China. The results indicate that the highest essential oil extraction rate was obtained from the pericarps (0.42%), primarily consisting of caryophyllene oxide (15.33%), nerolidol 2 (14.03%), and spathulenol (9.64%). This was followed by the leaves (0.21%), stems (0.09%), and roots (0.05%), with the highest content in their essential oils being caryophyllene (27.03%), cadina-1(10),4-diene (25.76%), and benzyl benzoate (17.11%), respectively. Hierarchical cluster analysis (HCA) and principal component analysis (PCA) revealed that, compared with the essential oils from stems and leaves, the essential oils from roots and pericarps showed relatively smaller differences and were usually clustered into one category. The leaf essential oil has the highest *in vitro* antioxidant activity, followed by the root, pericarp, and stem. This study aims to provide a scientific reference for the rational development and utilization of different parts of *Z. nitidum*, especially the leaf essential oil.

## 1. Introduction

*Zanthoxylum nitidum* (Roxb.) DC. is a perennial woody plant belonging to the Rutaceae family. It is primarily distributed in countries such as Vietnam, India, and Laos, and in the southern provinces of China, including Guangxi, Guangdong, Yunnan, Guizhou, Hunan, Fujian, and others [1,2]. Its dried roots are a traditional Chinese medicinal material and have been included in various editions of the Pharmacopoeia of the People’s Republic of China (PPRC). They possess properties such as anti-inflammatory, analgesic, antioxidant, antibacterial, and antitumor effects [3,4,5,6,7]. Currently, *Z. nitidum* is widely applied in the pharmaceutical industry as well as in daily-use products and cosmetics [4]. Its annual demand reaches 3000 tons, with annual sales exceeding RMB one billion [8]. *Z. nitidum* has become one of the natural plant resources with significant economic value in China.

As a perennial woody plant, *Z. nitidum* is typically harvested four to six years after planting, or even longer [7,8]. The PPRC records that only the roots of *Z. nitidum* are used for medicinal purposes [9], and its above-ground parts are usually discarded, leading to a significant waste of medicinal resources. Research has found that the types of components in the stems and roots of *Z. nitidum* are similar, with the main difference being in their content levels, where the alkaloid content in the above-ground parts is significantly lower than in the roots [10,11]. Pharmacological studies have indicated that the stem [11] and leaf [12] extracts of *Z. nitidum*, whether aqueous or alcoholic, also possess anti-inflammatory and analgesic pharmacological activities. To date, researchers have isolated and identified over 150 chemical components from different parts of *Z. nitidum*. These components include alkaloids, coumarins, lignans, flavonoids, terpenoids, and steroids, with the majority being alkaloids [4,7,13]. However, at present, there is only a limited amount of research on the essential oil composition of the fruits and leaves of *Z. nitidum* from Vietnam and India [14,15], and there are no studies on the composition of essential oils from different parts of *Z. nitidum* in China.

This study is the first to use Gas Chromatography–Mass Spectrometry (GC-MS) to identify and analyze the essential oil components extracted from different parts (roots, stems, leaves, and pericarps) of Chinese *Z. nitidum*, and to evaluate their *in vitro* antioxidant capacity. In addition, with the help of chemometrics methods, this study conducted hierarchical cluster analysis (HCA) and principal component analysis (PCA) on complex GC-MS data to classify and identify samples from different parts. The aim is to fill the gap in the literature on the chemical composition of essential oils from different parts of Chinese *Z. nitidum* and to provide a reference for the utilization of resources from different parts of *Z. nitidum*, especially the rational development and utilization of leaf essential oils.

## 2. Materials and Methods

### 2.1. Experimental Materials

**Figure 1:**
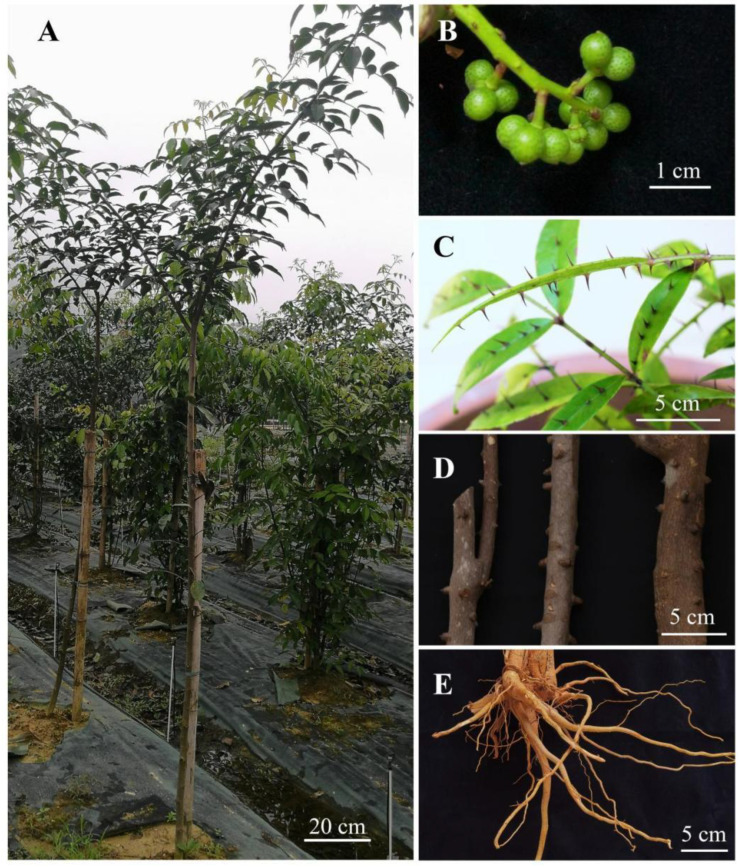
(**A**) *Zanthoxylum nitidum* planting base in Yunfu City, Guangdong Province, China. The (**B**) fruit, (**C**) leaves, (**D**) stems, and (**E**) roots of the *Z. nitidum*.

The different parts of *Z. nitidum* were collected in November 2022 from Yunfu City, Guangdong Province, China (112°3′ E, 22°54′ N) (Figure 1A). The samples were identified by Professor Wu Hong from South China Agricultural University as the fruits (Figure 1B), leaves (Figure 1C), stems (Figure 1D) and roots (Figure 1E) of the *Z. nitidum* belonging to the *Zanthoxylum* genus of the Rutaceae family. The samples were air-dried, crushed, sifted through a 60-mesh sieve, bagged, sealed, and stored in a desiccator.

### 2.2. Essential Oil Extraction and Content Determination

The 50 g samples (roots, stems, leaves, and pericarps) were weighed and placed separately into 1000 mL round-bottom flasks. Then, 400–500 mL of distilled water was added, shaken to mix, and soaked for 6 h. The essential oil determinator was connected to a reflux condenser. Water was added to the top of the condenser until it filled the scale part of the essential oil determinator and overflowed into the flask. The round-bottom flask was placed in an electric heating jacket and slowly heated to boiling (260 °C), maintaining a gentle boil for about 5 h. Heating was stopped when the amount of essential oil in the determinator no longer increased, and it was allowed to stand for a moment. After distillation, the effluent was transferred to a 250 mL separatory funnel, and the essential oil determinator was rinsed with dichloromethane three times, with the rinse collected in the separatory funnel. The upper aqueous layer was discarded, and the dichloromethane was transferred to a 250 mL conical flask. A small amount of anhydrous sodium sulfate was added, and the mixture was filtered. In a fume hood, the majority of the solvent was evaporated using a steam bath (55 °C). The concentrate was then transferred into a pre-dried collection tube and further distilled on the steam bath until all the dichloromethane was completely removed, resulting in a pale-yellow oily liquid. The oil was weighed (the weight difference before and after was the weight of the essential oil) and stored in 4 °C. Each sample was measured three times, and the final essential oil content was taken as the average value. The percentage of essential oil content was calculated as the weight of the essential oil divided by the weight of the dry sample powder. Variance analysis and plotting were performed in the Origin software (10.0), and differences were considered significant at the *p* < 0.05 level.

### 2.3. GC-MS Analysis

The gas chromatograph (7890A, Agilent, Santa Clara, CA, USA) coupled to the 5975C Plus mass spectrometer (Agilent, USA) was used for GC–MS analysis. The GC-MS operating conditions were as follows: The chromatographic column was a DB-5 MS (30 m × 0.25 µm × 0.25 mm), with a vaporization temperature of 270 °C. The temperature program started with an initial column temperature of 60 °C, held for 4 min, then ramped up to 150 °C at a rate of 8 °C/min, held at 150 °C for 10 min, ramped up to 200 °C at a rate of 5 °C/min, and finally ramped up to 280 °C at a rate of 10 °C/min. The carrier gas was high-purity helium with a flow rate of 1.0 mL/min. The essential oil was dissolved in dichloromethane to make a dilute solution, filtered through a 0.22 µm organic microporous membrane, and 2 µL was injected; the split ratio was 20:1; the solvent delay was 4 min. The EI ionization source was at 70 eV; the ion source temperature was 200 °C; the mass range scanned was *m*/*z* 35–650 amu. After mass spectrometry scanning of each peak in the total ion current chromatogram, the mass spectra were obtained. The compounds were identified using the NIST library search. The relative percentage content of each compound in the total essential oil was calculated by the peak area normalization method through the Xcalibur workstation data-processing system. The gas chromatogram covered a retention time range of 0–50 min. Each sample was measured three times, and the final relative content of compounds was taken as the average value.

### 2.4. Preparation of Essential Oil Solution

The essential oils extracted from different parts were dissolved in a 70% ethanol solution to prepare a test essential oil sample with a concentration of 0.5 mg/mL. The samples were stored at 4 °C for future use.

### 2.5. Total Antioxidant Capacity Determination (FRAP)

In this study, a commercial assay kit was used to determine the ferric reducing antioxidant power (FRAP), thereby measuring the antioxidant capacity. A total of 180 µL of the FRAP working solution was added to each well of a 96-well plate. Subsequently, 5 µL of the extract of each test essential oil sample or Trolox as a positive control was added, gently mixed, and incubated at 37 °C for 5 min. A multifunctional microplate reader (Infinite 200PRO, Tecan, Graz, Tyrol State, Austria) was used to measure the absorbance at OD_593_ nm. A standard curve was plotted, and the total antioxidant capacity of the samples was calculated based on the FeSO_4_·7H_2_O standard curve. The FeSO_4_·7H_2_O standard curve is shown in Figure S1. The experimental results indicated a good linear relationship between the concentration of FeSO_4_·7H_2_O and the absorbance values in the range of 0–2.5 mM, with the regression equation being y = 0.3806x + 0.009, *R*^2^ = 0.9986.

### 2.6. Total Antioxidant Capacity Determination (ABTS)

The decolorization analysis of the ABTS+ (2,2′-azino-bis(3-ethylbenzthiazoline-6-sulfonic acid)) radical cation was conducted according to the assay method of a commercial kit. In each well of a 96-well plate, 20 µL of the peroxidase working solution was added, followed by the addition of 10 µL of the test essential oil sample and thorough mixing. Finally, 170 µL of the ABTS working solution was added, mixed well, and left at room temperature for 6 min. The absorbance at the absorption wavelength of OD_734_ nm was measured. A Trolox standard curve was plotted, and the total antioxidant capacity of the samples was calculated based on the standard curve. Trolox, a water-soluble analog of vitamin E, was used as a reference standard for the preparation of the calibration curve. The Trolox standard curve is shown in Figure S2. The experimental results indicated a good linear relationship between the concentration of Trolox and the absorbance values in the range of 0–1.6 mM, with the regression equation being y = 0.9709x − 0.0677, *R*^2^ = 0.9939.

### 2.7. DPPH Radical Scavenging Activity Assay

The DPPH (1,1-diphenyl-2-picrylhydrazyl) radical scavenging activity was measured using a commercial assay kit in this study. In each well of a 96-well plate, 10 µL of the test essential oil samples from different parts of the *Z. nitidum* were added, followed by the addition of 190 µL of the DPPH solution. After the solution was thoroughly mixed, it was allowed to react for 30 min at 25 °C in the dark. The absorbance value (A) was measured at a wavelength of OD_515_ nm (Figure S3). The radical scavenging rate was calculated using the following formula: Scavenging rate % = (A0 − A) / A0 × 100% (where A0 is the value without the addition of the sample, and A is the value after the addition of the sample).

### 2.8. Hierarchical Clustering Analysis (HCA)

Hierarchical clustering is a method of cluster analysis designed to create a hierarchical structure of groups. Any suitable metric can be employed as a measure of similarity between pairs of observations. Consequently, samples within the same cluster exhibit significant similarities, while those in different clusters display marked differences [16]. In this study, hierarchical cluster analysis and Pearson’s correlation were utilized. The relative peak areas in GC-MS were selected as the measurement values for cluster analysis to construct the distance matrices for chromatographic area and samples, respectively. The cluster analysis and dendrogram were performed using SPSS version 20.0.

### 2.9. Principal Component Analysis (PCA)

PCA was performed using the relative peak areas obtained from GC-MS analysis. The initial scores derived from PCA were utilized to construct a projection plot, which visually depicted the similarities among the fingerprints. In this study, the PCA analysis was conducted using SPSS version 20.0 [16].

### 2.10. Statistical Analysis

All values were presented as mean ± SEM. Data analysis was performed using one-way analysis of variance (ANOVA), followed by Duncan’s multiple comparison test. Statistical analysis was conducted using SPSS (version 20.0; IBM, New York, NY, USA). The *p* < 0.05 were considered statistically significant.

## 3. Results and Discussion

### 3.1. Comparison of Essential Oil Content

**Figure 2:**
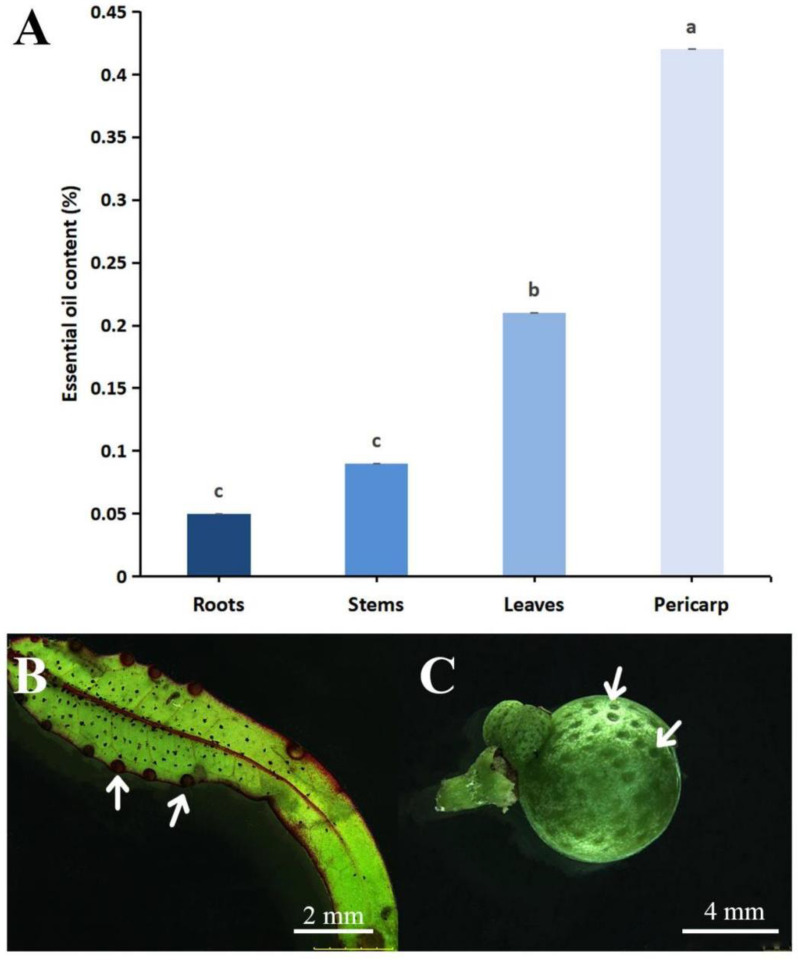
(**A**) Essential oil extraction rates from different parts of *Zanthoxylum nitidum* on a dry weight basis. Different letters indicate statistically significant differences between samples (*p* < 0.05). Secretory cavities (white arrows) on the (**B**) leaves and (**C**) pericarp of *Z. nitidum*.

The essential oil content in the roots, stems, leaves, and pericarps of *Z. nitidum* shows significant differences (Figure 2A). The essential oil content in the pericarp (0.42%) was significantly higher than in other parts (*p* < 0.05), followed by that of the leaves (0.21%). There was no significant difference in the essential oil content between the stem (0.09%) and the root (0.05%) (*p* > 0.05). Among plants of the same family, the essential oil contents in the fruit pericarps of *Citrus sinensis* (L.) Osbeck, *C. × aurantium* Siebold & Zucc. ex Engl., and *C. reticulata* Blanco were all higher than that in the leaves [17,18], which is consistent with the results of our study. Secretory cavities, an important anatomical feature of Rutaceae family plants, are the primary storage site for essential oils [19,20,21,22]. We found secretory cavities in *Z. nitidum* leaf margins (Figure 2B), consistent with Liu and Hu’s [19] findings. These leaf secretory cavities are larger but fewer in number. Moreover, more secretory cavities were found in the fruit peel (Figure 2C), explaining the significantly higher essential oil content in the pericarp than in the leaves. In addition, the stem has relatively fewer secretory cavities, and the roots typically lack secretory cavities [19,20,23]. Therefore, compared to the roots and stems, the pericarps and leaves often contain a higher amount of essential oils.

### 3.2. Chemical Composition

**Figure 3:**
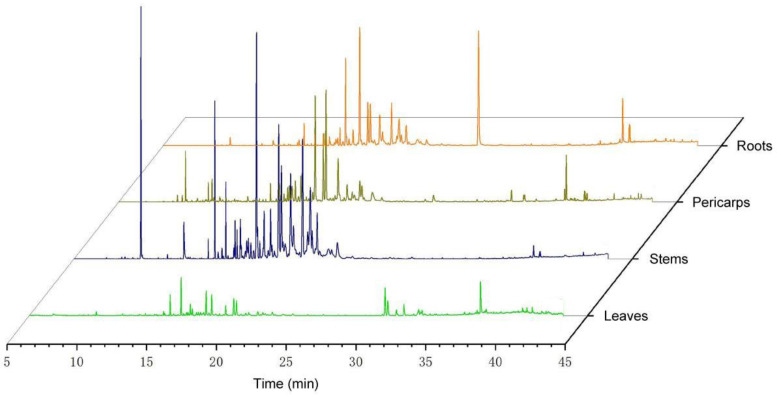
The GC-MS profiles of different parts of *Zanthoxylum nitidum*.

In the *Z. nitidum* essential oils from roots, stems, leaves, and pericarps, 163 compounds were identified. Of these, 57 compounds had relative contents over 1% (Figure 3; Table S1). In the essential oil of the root, a total of 32 components were identified, with the main constituents being benzyl benzoate (17.11%), nerolidol 2 (11.30%), cadina-1(10),4-diene (7.16%), di-epi-1,10-cubenol (5.37%), and τ-muurolol (4.98%). The stem essential oil was found to contain 20 components, with the main constituents being cadina-1(10),4-diene (25.76%), linalool (18.81%), copaene (10.90%), caryophyllene (6.10%), and geraniol (4.61%). The leaf essential oil contained 19 components, with the main constituents being caryophyllene (27.03%), α-cubebene (15.59%), humulene (9.89%), (e)-2-epi-β-caryophyllene (5.83%), and octadecyl iodide (4.28%). The pericarp essential oil had 29 components identified, with the main constituents being caryophyllene oxide (15.33%), nerolidol 2 (14.03%), spathulenol (9.64%), humulene epoxide II (6.83%), and 1h-cyclopropa[a]naphthalene, 1a,2,3,3a,4,5,6,7b-octahydro-1,1,3a,7-tetramethyl-, [1aR-(1aα,3aα,7bα)]- (3.60%).

The same medicinal plant will synthesize and accumulate different types and amounts of secondary metabolites in different growth environments and various tissue organs [24,25]. Research has found that the essential oils from different parts of *Z. nitidum* are primarily composed of sesquiterpenes, with the main differences being in their content. The content of sesquiterpenes in the roots, stems, leaves, and pericarps was 57.12%, 56.91%, 68.5%, and 71.6%, respectively. Following these were monoterpene compounds, accounting for 1.28%, 23.87%, 3.99%, and 5.38%, respectively. The production and distribution of plant secondary metabolites typically exhibit specificity related to the genus, organ tissue, and growth and development stage [26]. Different parts of a plant perform various physiological functions, and the cells in different parts may contain different enzymes and metabolic pathways, leading to the synthesis of different secondary metabolites. For instance, there are significant differences in the types and content of secondary metabolites in different parts of plants such as *Centella asiatica* (L.) Urb., *Hypericum orientale* L., and *Z. nitidum* [16,27,28].

In contrast to Chinese *Z. nitidum*, the main components of the essential oils from the fruits, leaves, and stems of Vietnamese *Z. nitidum* are alkanes (46.6%), monoterpenes (62.1%), and non-terpenoid cyclic ketones (72.8%), respectively [15]. In Indian *Z. nitidum*, the essential oils from both the fruits and leaves are predominantly composed of monoterpenes, accounting for 75% and 60%, respectively [14]. In Vietnam and India, the main components of *Z. nitidum* leaf essential oils are monoterpenes, while in this study, the leaf essential oil was mainly sesquiterpenes (68.5%). The main components of the Vietnamese leaf essential oil were limonene (44.3%), β-caryophyllene (12.5%), and linalool (11.0%) [15]. For the Indian one, they were limonene (33.1%), geraniol (10.6%), and carvone (9.6%) [14]. The most abundant component in both was limonene. In contrast, the most abundant compounds in the Chinese leaf essential oil were caryophyllene (27.03%), α-cubebene (15.59%), and humulene (9.89%). It is well known that in different growth environments, the differences in secondary metabolites are mainly due to the significant impact of environmental factors on plant physiological and biochemical reactions, as well as the secondary metabolic processes [25,29]. Environmental stress, including abiotic and biotic factors such as drought, salinity, cold, heat, ultraviolet radiation (UVr), reactive oxygen species (ROS), trace metals (TM), and soil pH, can lead to physiological and biochemical changes in plants, thereby affecting the accumulation of secondary metabolites [25,30]. Research has found that the content of main alkaloids in the roots of *Z. nitidum* from Guangdong province was significantly higher than that from the Guangxi province production area [16]. Meanwhile, the content of naringin and total flavonoids in *Z. nitidum* from Qujing City, Yunnan Province, is higher than that in Guangdong and Guangxi provinces [6]. Additionally, the production and alteration of plant secondary metabolites are one of the important mechanisms for plants to adapt to environmental changes. When plants perceive changes in external environmental factors, they trigger a series of signal transduction cascades that activate or inhibit the expression of downstream genes, thereby regulating plant growth and development processes and changes in metabolites [30,31]. Therefore, medicinal plants such as *Z. nitidum* and *Cinnamomum cassia* (L.) D. Don, which are geographically close or have similar growth environments, tend to have more similar types and contents of secondary metabolites, often clustering into a category [16,32].

### 3.3. HCA and PCA of Z. nitidum

**Figure 4:**
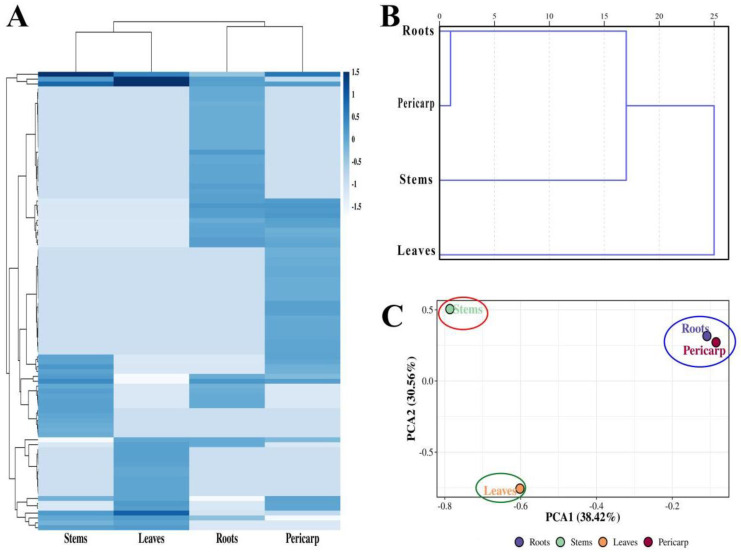
The components of different parts of *Zanthoxylum nitidum* (**A**) heatmap, (**B**) hierarchical cluster analysis (HCA), and (**C**) principal component analysis (PCA).

Chemometrics is a highly useful tool in the quality control of traditional Chinese herbal medicine. It employs mathematics, statistics, and other methods to maximize the extraction of information from data obtained through various analytical techniques [33,34]. To more intuitively observe the relationships between the essential oil components of different parts of *Z. nitidum*, we performed cluster analysis using the peak areas of all components from the GC-MS analysis of essential oils from different parts (Figure 4A). We found that the similarity in the types and contents of essential oil components among the four parts was relatively low. Subsequently, the data were normalized and imported into SPSS 20.0 software, where HCA was performed using the median clustering method and the block method (Figure 4B). When the critical value was within 5, the roots and pericarps clustered together first. When the critical value was between 15 and 20, they clustered with the stems. It was only when the critical value reached 25 that they clustered with the leaves. HCA analysis revealed significant similarity between the composition of the essential oils in the root and pericarp.

HCA can accurately classify objects at various distance levels, but it cannot explicitly display the relationships between non-adjacent research objects [16,35]. To more clearly elucidate the relationships among the research subjects, this study established a PCA model using the peak areas of all components from the GC-MS analysis of essential oils from different parts as variables. PCA is a statistical method that projects the original high-dimensional data onto a lower-dimensional space through linear transformation, retaining the directions with the maximum variance, thereby achieving the goal of data dimensionality reduction [2,33,34]. Figure 4C was a two-dimensional scatter plot of PC1 and PC2 obtained from the PCA of essential oils from different parts. The variance contribution rate of the PC1 was 38.42%, and that of the PC2 was 30.56%. The cumulative variance contribution rate of the first two principal components was 68.98%. Therefore, the first two principal components were essentially capable of reflecting more than half of the original spectral information of the samples. In the principal component space, the various samples were positioned relatively independently, and samples from different parts were effectively distinguished from each other. Figure 4C showed that the positional relationships of essential oils from different parts of *Z. nitidum* on the two-dimensional ordination plot corresponded to a certain extent with their similarities. Similar to the results of cluster analysis, the roots and pericarps were positioned relatively close to each other on the two-dimensional plot and clustered into one group, indicating that their essential oil contents were lower different compared to those of the stems and leaves. Numerous studies have found that the content of components in different parts of traditional Chinese medicinal materials such as *Nauclea officinalis* (Pierre ex Pit.) Merr. & Chun, *C. cassia*, *Panax ginseng* C. A. Mey. and *Panax notoginseng* (Burkill) F. H. Chen ex C. H. Chow varies greatly [36,37,38]. Different parts can be effectively clustered in the analysis. For example, the root bark, stem bark, and branch bark of *N. officinalis* cluster into one category, while the root wood, stem wood, and small branches cluster into another category, and the leaves form a separate category on their own [37].

### 3.4. In Vitro Antioxidant Ability

**Figure 5:**
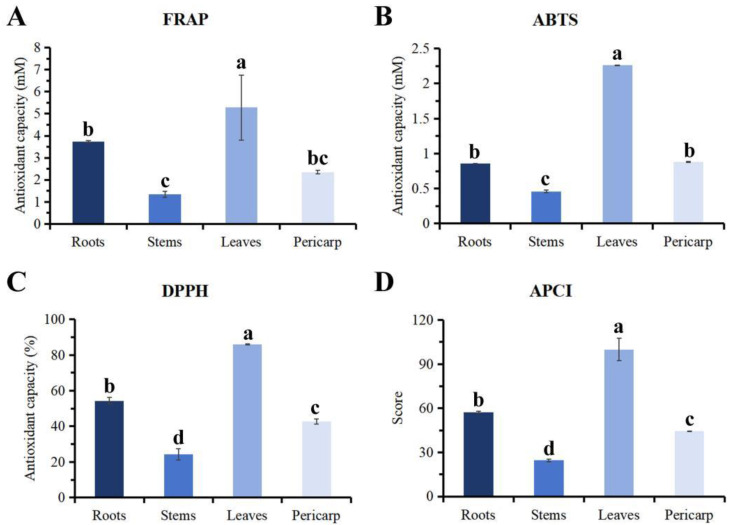
The (**A**) ferric reducing antioxidant power (FRAP), (**B**) the total antioxidant capacity of ABTS+ (2,2′-azino-bis(3-ethylbenzthiazoline-6-sulfonic acid)), (**C**) the DPPH (1,1-diphenyl-2-picrylhydrazyl) radical scavenging activity, and (**D**) the antioxidant potency composite index (APCI) of different parts of *Zanthoxylum nitidum*. The different letters indicate significant difference (*p* < 0.05).

Research has found that the differences in antioxidant activity of essential oils from different parts, as measured by DPPH, FRAP, and ABTS, are relatively small (Figure 5, Table 1). The overall results show that the essential oil from the leaves has the strongest *in vitro* antioxidant capacity, followed by that from the roots. In order to more clearly obtain the ranking of the total antioxidant capacity of different parts, this experiment used the antioxidant potency composite index (APCI) [6] to evaluate the antioxidant capacity of different parts of *Z. nitidum* (Table 1). The results indicate that the APCI of the leaf essential oil is the highest (100). This is followed by the root (57.37) and pericarp (44.38) (*p* > 0.05). The APCI of the stem essential oil is the lowest (24.63).

The monoterpenes and sesquiterpenes found in essential oils both possess anti-inflammatory, antioxidant, antitumor, and antimicrobial properties [39,40,41]. Among them, Caryophyllene, as a free radical scavenger, can inhibit lipid peroxidation and has strong scavenging activity against hydroxyl radicals and superoxide anions [42]. This study found that the content of Caryophyllene in the leaf essential oil (27.03%) was significantly higher than in other parts, which may also be the main reason why the comprehensive antioxidant capacity evaluation index of the leaf essential oil is higher than that of other parts.

## 4. Conclusions

There are differences in the extraction rates, chemical compositions, and *in vitro* antioxidant capacities of the essential oils from different parts of *Z. nitidum*. The pericarp essential oil has the highest extraction rate (0.42%), followed by the leaves (0.21%), while the essential oils from the stem (0.09%) and root (0.05%) have the lowest extraction rates. A total of 57 main components were identified in the essential oils from different parts of *Z. nitidum*, with sesquiterpenes being the predominant group. The pericarp essential oil had 29 components, mainly caryophyllene oxide (15.33%), nerolidol 2 (14.03%), spathulenol (9.64%), and humulene epoxide II (6.83%). The roots’ essential oil contained 32 components, primarily benzyl benzoate (17.11%), nerolidol 2 (11.30%), and cadina-1(10),4-diene (7.16%). The stem essential oil had 20 components, with the main ones being cadina-1(10),4-diene (25.76%), linalool (18.81%), and copaene (10.90%). The leaves’ essential oil had 19 components, mainly caryophyllene (27.03%), α-cubebene (15.59%), and humulene (9.89%). HCA and PCA revealed that, compared with the essential oils from stems and leaves, the essential oils from roots and pericarps showed relatively smaller differences and were usually clustered into one category. In *in vitro* antioxidant studies, the leaf essential oil had the highest APCI score (100), followed by the root (57.37) and pericarp (44.38), with the stem presenting the lowest score (24.63). The results of this research fill the gap in the literature on the chemical composition of essential oils from different parts of Chinese *Z. nitidum* and provide a reference for the rational development and utilization of the resources from different parts, especially the leaf essential oil.

## Figures and Tables

**Table 1:** *In vitro* antioxidant activities of essential oils from different parts of *Zanthoxylum nitidum*.

Samples	FRAP (mM)	ABTS (mM)	DPPH (%)	Antioxidant Potency Composite Index (APCI)	Sort
Roots	3.74 ± 0.042 ^b^	0.86 ± 0.0012 ^b^	54.36 ± 1.85 ^b^	57.37 ± 0.79 ^b^	2
Stems	1.34 ± 0.13 ^c^	0.46 ± 0.018 ^c^	24.21 ± 3.15 ^d^	24.63 ± 0.91 ^d^	4
Leaves	5.28 ± 1.47 ^a^	2.26 ± 0.0061 ^a^	85.95 ± 0.26 ^a^	100.00 ± 7.67 ^a^	1
Pericarp	2.35 ± 0.082 ^bc^	0.88 ± 0.0092 ^b^	42.72 ± 1.38 ^c^	44.38 ± 0.29 ^c^	3

Note: Different letters indicate significant difference (*p* < 0.05).

## Data Availability

Data are contained within the article and Supplementary Materials.

## Supplementary Materials

The following supporting information can be downloaded at: https://www.mdpi.com/article/10.3390/plants14081194/s1, Figure S1: Standard curve of FeSO_4_·7H_2_O; Figure S2: Standard curve of Trolox; Figure S3: Standard curve of Gallic Acid; Table S1: Chemical constituents of essential oils from different parts of *Zanthoxylum nitidum*.

## References

[1] Editorial Committee of Flora of China (2007). Chinese Academy of Sciences, Flora of China.

[2] Yang Y., He J., Liu Y., Zeng J., Zeng L., He R., Guiang M.M., Li Y., Wu H. (2023). Assessment of Chinese suitable habitats of *Zanthoxylum nitidum* in different climatic conditions by Maxent model, HPLC, and chemometric methods. Ind. Crop Prod..

[3] Lin Q., Pu H., Guan H., Ma C., Zhang Y., Ding W., Cheng X., Ji L., Wang Z., Wang C. (2020). Rapid identification and pharmacokinetic studies of multiple active alkaloids in rat plasma through UPLC-Q-TOF-MS and UPLC-MS/MS after the oral administration of *Zanthoxylum nitidum* extract. J. Pharm. Biomed..

[4] Lu Q., Ma R., Yang Y., Mo Z., Pu X., Li C. (2020). *Zanthoxylum nitidum* (Roxb.) DC: Traditional uses, phytochemistry, pharmacological activities and toxicology. J. Ethnopharmacol..

[5] Qin F., Wang F.-F., Wang C.-G., Chen Y., Li M.-S., Zhu Y.-K., Huang X.-C., Fan C.-W., Wang H.-S. (2021). The neurotrophic and antineuroinflammatory effects of phenylpropanoids from *Zanthoxylum nitidum* var. *tomentosum* (Rutaceae). Fitoterapia.

[6] He J., Zeng J., Zeng L., Yang L., Ma Q., Wu H., Yang Y. (2023). Comparison of the bioactive components and antioxidant activities of wild-type *Zanthoxylum nitidum* roots from various regions of Southern China. Nat. Prod. Res..

[7] Liu Y., Yang Y., Jia H., Tan P., Zeng J., Zeng L., Xie T., Geng X., He H., Bai M. (2024). A comparative analysis of the composition of the primary bioactive molecules, antioxidant activities, and anti-inflammation abilities of the roots of *Zanthoxylum nitidum* harvested at different years of cultivation. Ind. Crop Prod..

[8] Peng Z.H., Wu M.H., Xie Z.J., Yang X.B., Lai M.X. (2018). Investigation of wild resource of *Zanthoxylum nitidum*. Pharm. Today.

[9] China Pharmacopeia Commission (2020). Pharmacopoeia of the People’s Republic of China (I).

[10] He L.L., Lin Z.H., Hu Y.Z., Qin Q.Y., Huang G.W., Hu D.N. (2013). Dynamic accumulation research of active ingredient in different part and growth phase of caltivative *Zanthoxylum nitidum* by UPLC-DAD. Chin. J. Exp. Tradit. Med. Formula.

[11] Ma Q., Wu G.S., Zheng L.T., Han Z.Z., Zhan R.T., Chen W.W., Niu M. (2020). UPLC specific chromatogram study and pharmacodynamic composition analysis of roots and stems of *Zanthoxylum nitidum*. J. Chin. Med. Mater..

[12] Mackhaphonh V. (2013). Studies on the Anti-Inflammatory and Analgesic Active of Different Portions and Low-Polarity Portion Chemical Constituents in the Leaves of Zanthoxylum nitidum DC.

[13] Ali F., Alom S., Zaman M.K. (2022). Ethnobotany, phytochemistry and pharmacological properties of *Zanthoxylum nitidum*: A systemic review. World J. Pharm. Res..

[14] Bhattacharya S., Zaman M.K. (2009). Essential oil composition of fruits and leaves of *Zanthoxylum nitidum* grown in upper Assam region of India. Pharmacogn. Res..

[15] Tuyen T.T., Quan P.M., Thu Le V.T., Toan T.Q. (2021). Chemical composition, antimicrobial, and cytotoxic activities of leaf, fruit, and branch essential oils obtained from *Zanthoxylum nitidum* grown in Vietnam. Nat. Prod. Commun..

[16] Yang Y., Li Y., Amoroso V., Acma F., Guiang M.M., Wu H. (2023). Comparison of production of bioactive components in *Zanthoxylum nitidum* taproots from different regions in southern China. Biomed. Chromatogr..

[17] Chen L.H. (2018). Research on the Extraction, Antibacterial and Insecticidal Activities of Citrus Essential Oil.

[18] Okla M.K., Alamri S.A., Salem M.Z., Ali H.M., Behiry S.I., Nasser R.A., Alaraidh I.A., Al-Ghtani S.M., Soufan W. (2019). Yield, phytochemical constituents, and antibacterial activity of essential oils from the leaves/twigs, branches, branch wood, and branch bark of sour orange (*Citrus aurantium* L.). Processes.

[19] Liu W.Z., Hu Z.H. (1998). Comparative anatomy of secretory cavities in leaves of the rutaceae in China. Acta Phytotax Sin..

[20] Liu W.Z., Hu Z.H. (1999). Studies on the secretory cavity of stems in Rutaceae. Acta Bot. Boreal. Occident. Sin..

[21] Singh B., Singh J.P., Kaur A., Yadav M.P. (2021). Insights into the chemical composition and bioactivities of citrus peel essential oils. Food Res. Int..

[22] Salvatore M.M., Nicoletti R., Andolfi A. (2022). Essential oils in *Citrus* fruit ripening and postharvest quality. Horticulturae.

[23] Metcalfe G.R., Chalk L. (1950). Anatomy of the Dicotyledons.

[24] Figueiredo A.C., Barroso J.G., Pedro L.G., Scheffer J.J.C. (2008). Factors affecting secondary metabolite production in plants: Volatile components and essential oils. Flavour Frag. J..

[25] Li Y., Kong D., Fu Y., Sussman M.R., Wu H. (2020). The effect of developmental and environmental factors on secondary metabolites in medicinal plants. Plant Physiol. Biochem..

[26] Bhatla S.C., Lal M.A. (2023). Secondary Metabolites. Plant Physiology, Development and Metabolism.

[27] Cirak C., Radusiene J., Stanius Z., Camas N., Caliskan O., Odabas M.S. (2012). Secondary metabolites of *Hypericum orientale* L. growing in Turkey: Variation among populations and plant parts. Acta Physiol. Plant.

[28] Biradar S.R., Rachetti B.D. (2013). Extraction of some secondary metabolites & thin layer chromatography from different parts of *Centella asiatica* L. (URB). Am. J. Life Sci..

[29] Verma N., Shukla S. (2015). Impact of various factors responsible for fluctuation in plant secondary metabolites. J. Appl. Res. Med. Aromat. Plants.

[30] Qaderi M.M., Martel A.B., Strugnell C.A. (2023). Environmental factors regulate plant secondary metabolites. Plants.

[31] Neugart S., Baldermann S., Hanschen F.S., Klopsch R., Wiesner-Reinhold M., Schreiner M. (2018). The intrinsic quality of brassicaceous vegetables: How secondary plant metabolites are affected by genetic, environmental, and agronomic factors. Sci. Hortic..

[32] Li Y.-Q., Kong D.-X., Huang R.-S., Liang H.-L., Xu C.-G., Wu H. (2013). Variations in essential oil yields and compositions of *Cinnamomum cassia* leaves at different developmental stages. Ind. Crop Prod..

[33] Huang Y., Wu Z., Su R., Ruan G., Du F., Li G. (2016). Current application of chemometrics in traditional Chinese herbal medicine research. J. Chromatogr. B.

[34] Ding R., Yu L., Wang C., Zhong S., Gu R. (2023). Quality assessment of traditional Chinese medicine based on data fusion combined with machine learning: A review. Crit. Rev. Anal. Chem..

[35] Zhang Y., Wang Z.-Z. (2008). Comparative analysis of essential oil components of three *Phlomis* species in Qinling mountains of China. J. Pharm. Biomed..

[36] Chen P., Yu J., Lu F., Lin M., Cheng H. (2016). Differentiating parts of *Cinnamomum cassia* using LC-qTOF-MS in conjunction with principal component analysis. Biomed. Chromatogr..

[37] Peng J., Fan X.Y., Wang D.L., Xu B., Zhang Z.K., Xiao W.Q., Yang T.Z., Li P.Y., Du S.Y. (2023). Differences of *Nauclea officinalis* in different parts based on quantitative analysis of components and fingerprint by chemical pattern recognition. Chin. Tradit. Herb. Drug.

[38] Zhang F., Han X., Li D., Liu Z. (2024). Differences of malonyl ginsenosides and neutral ginsenosides in different parts of *Panax ginseng* and *Panax notoginseng* by high performance liquid chromatography and chemometric analysis. China Food Addit..

[39] Yang W., Chen X., Li Y., Guo S., Wang Z., Yu X. (2020). Advances in pharmacological activities of terpenoids. Nat. Prod. Commun..

[40] Baccouri B., Rajhi I. (2021). Chapter 5: Potential antioxidant activity of terpenes. Terpenes and Terpenoids: Recent Advances.

[41] Kokilananthan S., Bulugahapitiya V.P., Manawadu H., Gangabadage C.S. (2022). Sesquiterpenes and monoterpenes from different varieties of guava leaf essential oils and their antioxidant potential. Heliyon.

[42] Calleja M.A., Vieites J.M., Montero-Meterdez T., Torres M.I., Faus M.J., Gil A., Suárez A. (2013). The antioxidant effect of β-caryophyllene protects rat liver from carbon tetrachloride-induced fibrosis by inhibiting hepatic stellate cell activation. Br. J. Nutr..

