# Supplementary material for: Comparison of Essential Oil Components and In Vitro Antioxidant Activity of Zanthoxylum nitidum from Different Parts
Source: Plants (Basel). 2025 Apr 11;14(8):1194. doi: 10.3390/plants14081194 (PMC12029993; doi:10.3390/plants14081194)
Supplement: Supplementary file 1 [file plants-14-01194-s001.zip › plants-3524235-supplementary.pdf]

Supplementary Materials

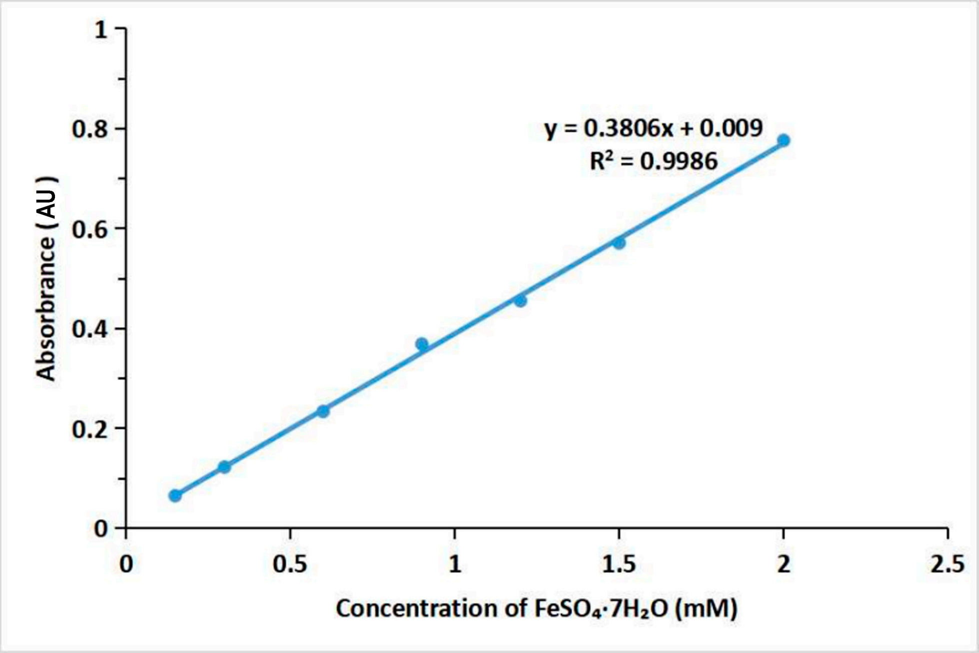

Figure S1 Standard curve of $FeSO_4 \cdot 7H_2O$.

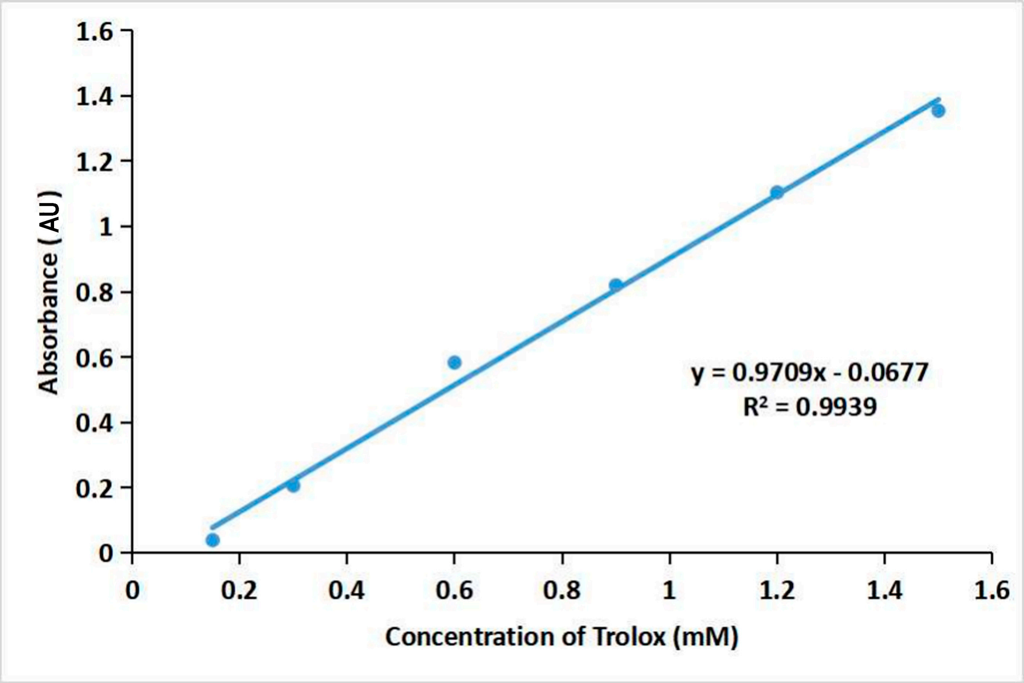

Figure S2 Standard curve of Trolox.

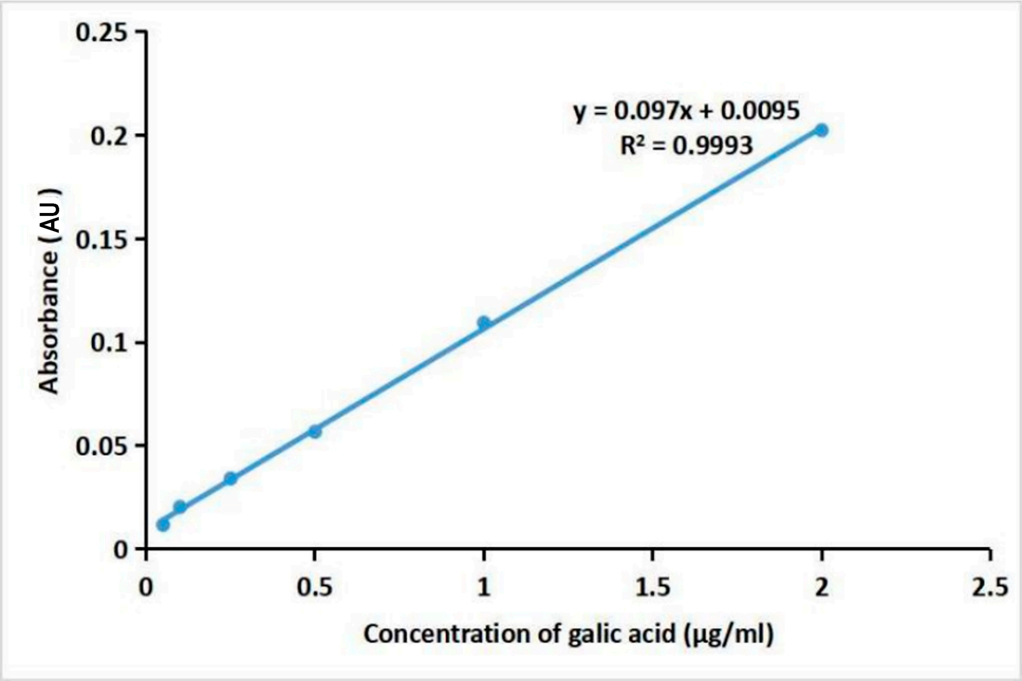

Figure S3 Standard curve of Gallic Acid.

Table S1 Chemical constituents of essential oils from different parts of *Zanthoxylum nitidum*.

No.	Compounds	Relative content (%)				Identification
		Roots	Stems	Leaves	Pericarp	
1	5-Methyl furfural	-	-	3.10 ± 0.21^{a}	-	GC–MS,RT
2	Linalool	0.44 ± 0.029^{c}	18.81 ± 0.85^{a}	2.69 ± 0.086^{b}	3.57 ± 0.39^{b}	GC–MS,RT
3	(-)-Terpinen-4-ol	-	-	-	1.51 ± 0.061	GC–MS,RT
4	α-Terpineol	-	0.31 ± 0.057^{b}	1.30 ± 0.19^{a}	1.81 ± 0.30^{a}	GC–MS,RT
5	Geraniol	0.45 ± 0.019^{b}	4.61 ± 0.24^{a}	-	-	GC–MS,RT
6	Lavandulal	-	-	1.34 ± 0.21^{a}	-	GC–MS,RT
7	2,3,5-Trimethyldecane	-	0.13 ± 0.046^{b}	1.76 ± 0.14^{a}	-	GC–MS,RT
8	3-Methoxyacetophenone	-	-	1.70 ± 0.16^{a}	-	GC–MS,RT
9	1,1,6-Trimethyl-1,2-dihydronaphthalene	-	-	1.27 ± 0.20^{a}	-	GC–MS,RT
10	α-Cubebene	1.27 ± 0.034^{b}	1.34 ± 0.080^{b}	15.59 ± 0.56^{a}	0.20 ± 0.090^{c}	GC–MS,RT
11	Copaene	-	10.90 ± 0.92^{a}	-	0.27 ± 0.056^{b}	GC–MS,RT
12	β-Copaene	0.05 ± 0.0042^{c}	0.93 ± 0.18^{b}	3.38 ± 0.27^{a}	-	GC–MS,RT
13	Caryophyllene	1.22 ± 0.056^{c}	6.10 ± 0.18^{b}	27.03 ± 1.01^{a}	1.40 ± 0.12^{c}	GC–MS,RT
14	2,4,6-Trimethylquinoline	-	-	3.51 ± 0.15^{a}	-	GC–MS,RT
15	Squalene	-	-	2.91 ± 0.13^{a}	-	GC–MS,RT
16	Humulene	-	-	9.89 ± 0.68^{a}	1.56 ± 0.19^{b}	GC–MS,RT
17	1,4,7,-Cycloundecatriene, 1,5,9,9-tetramethyl-, Z,Z,Z-	0.77 ± 0.016^{b}	3.41 ± 0.080^{a}	-	-	GC–MS,RT
18	β-D-Mannofuranoside, O-geranyl	-	-	1.69 ± 0.12^{a}	-	GC–MS,RT
19	(E)-2-epi-β-caryophyllene	-	2.76 ± 0.14^{b}	5.83 ± 0.15^{a}	-	GC–MS,RT
20	Naphthalene, 1,2,3,4,5,6-hexahydro-4,7-dimethyl-1-(1-methylethyl)-	0.64 ± 0.046^{b}	3.67 ± 0.14^{a}	-	0.12 ± 0.07^{c}	GC–MS,RT
21	γ-Muurolene	0.28 ± 0.033^{c}	1.24 ± 0.056^{b}	2.88 ± 0.18^{a}	0.37 ± 0.13^{c}	GC–MS,RT

22	Germacrene D	-	-	4.14 ± 0.22[a]	1.49 ± 0.073[b]	GC–MS,RT
23	β-Selinene	-	-	-	1.75 ± 0.16[a]	GC–MS,RT
24	Octadecyl iodide	-	-	4.28 ± 0.23[a]	-	GC–MS,RT
25	Bicyclosesquiphellandrene	-	3.00 ± 0.11[a]	-	-	GC–MS,RT
26	α-Selinene	0.40 ± 0.041[b]	-	-	2.79 ± 0.69[a]	GC–MS,RT
27	α-Muurolene	0.66 ± 0.029[b]	2.40 ± 0.098[a]	-	-	GC–MS,RT
28	2,5-Di-tert-butylphenol	1.46 ± 0.049[a]	-	-	-	GC–MS,RT
29	2,4-Di-tert-butylphenol	-	1.98 ± 0.19[b]	-	2.26 ± 0.30[a]	GC–MS,RT
30	(+)-γ-cadinene	-	1.59 ± 0.11[a]	-	-	GC–MS,RT
31	Bicyclo[4.4.0]dec-1-ene, 2-isopropyl-5-methyl-9-methylene-	0.57 ± 0.054[b]	-	3.14 ± 0.15[a]	-	GC–MS,RT
32	Cadina-1(10),4-diene	7.16 ± 0.12[b]	25.76 ± 1.45[a]	-	2.97 ± 0.16[c]	GC–MS,RT
33	Epizonarene	0.73 ± 0.033[b]	3.08 ± 0.19[a]	-	-	GC–MS,RT
34	Naphthalene, 1,2,3,4,4a,7-hexahydro-1,6-dimethyl-4-(1-methylethyl)-	-	2.21 ± 0.22[a]	-	0.24 ± 0.074[b]	GC–MS,RT
35	α-Calacorene	1.78 ± 0.13[a]	-	-	0.36 ± 0.050[b]	GC–MS,RT
36	(2Z,4E)-3,7,11-trimethyldodeca-2,4,10-triene	-	-	-	1.23 ± 0.14[a]	GC–MS,RT
37	Nerolidol 2	11.30 ± 0.43[b]	-	-	14.03 ± 0.17[a]	GC–MS,RT
38	Spathulenol	4.84 ± 0.14[b]	-	-	9.64 ± 0.57[a]	GC–MS,RT
39	Caryophyllene oxide	4.71 ± 0.094[b]	-	-	15.33 ± 0.49[a]	GC–MS,RT
40	1H-Cycloprop[e]azulen-4-ol, decahydro-1,1,4,7-tetramethyl-, [1aR-(1aα,4β,4aβ,7α,7aβ,7bα)]-	1.05 ± 0.075[a]	-	-	-	GC–MS,RT
41	Ledol	4.34 ± 0.10[a]	-	-	0.81 ± 0.085[b]	GC–MS,RT
42	Humulene epoxide II	2.48 ± 0.19[b]	-	-	6.83 ± 0.19[a]	GC–MS,RT
43	Valerena-4,7(11)-diene	-	1.24 ± 0.18[a]	-	1.05 ± 0.18[a]	GC–MS,RT
44	Di-epi-1,10-cubenol	5.37 ± 0.24[a]	-	-	-	GC–MS,RT
45	Isospathulenol	-	-	-	3.11 ± 0.15[a]	GC–MS,RT

46	(-)-Spathulenol	1.38 ± 0.11[b]	-	-	1.80 ± 0.65[a]	GC–MS,RT
47	τ-Muurolol	4.98 ± 0.16[a]	-	-	1.56 ± 0.14[b]	GC–MS,RT
48	Cadinol	1.55 ± 0.11[a]	-	-	0.52 ± 0.070[b]	GC–MS,RT
49	1H-Cyclopropa[a]naphthalene, 1a,2,3,3a,4,5,6,7b-octahydro-1,1,3a,7-tetramethyl-, [1aR-(1aα,3aα,7bα)]-	-	-	-	3.60 ± 0.21[a]	GC–MS,RT
50	α-Cadinol	2.83 ± 0.066[a]	-	-	-	GC–MS,RT
51	Neointermedeol	-	-	-	3.10 ± 0.37[a]	GC–MS,RT
52	Cycloheptane, 1-ethenyl-1-methyl-4-methylene-2-(2-methyl-1-propenyl)-	1.61 ± 0.086[a]	-	-	-	GC–MS,RT
53	5-cyclodecen-1-ol, 4,10-bis(methylene)-7-(1-methylethyl)-, (1R,5E,7S)-	1.32 ± 0.067[a]	-	-	-	GC–MS,RT
54	Benzyl Benzoate	17.11 ± 0.30[a]	-	-	-	GC–MS,RT
55	Incensole	3.71 ± 0.17[a]	-	-	-	GC–MS,RT
56	2,5-Dimethylcyclohexanol	1.32 ± 0.079[a]	-	-	-	GC–MS,RT
57	palmitic acid	1.07 ± 0.090[a]	-	-	-	GC–MS,RT
Total		88.85	95.47	97.43	85.28	GC–MS,RT

Note: -: Not detected. Data is expressed as mean ± SEM. Bars sharing the same small letter within a line did not share significant differences at $P < 0.05$.
